# Author Correction: A pangenome and pantranscriptome of hexaploid oat

**DOI:** 10.1038/s41586-025-09919-7

**Published:** 2025-11-20

**Authors:** Raz Avni, Nadia Kamal, Lidija Bitz, Eric N. Jellen, Wubishet A. Bekele, Tefera T. Angessa, Petri Auvinen, Oliver Bitz, Brian Boyle, Francisco J. Canales, Craig H. Carlson, Brett Chapman, Harmeet Singh Chawla, Yutang Chen, Dario Copetti, Samara Correia de Lemos, Viet Dang, Steven R. Eichten, Kathy Esvelt Klos, Amit M. Fenn, Anne Fiebig, Yong-Bi Fu, Heidrun Gundlach, Rajeev Gupta, Georg Haberer, Tianhua He, Matthias H. Herrmann, Axel Himmelbach, Catherine J. Howarth, Haifei Hu, Julio Isidro y Sánchez, Asuka Itaya, Jean-Luc Jannink, Yong Jia, Rajvinder Kaur, Manuela Knauft, Tim Langdon, Thomas Lux, Sofia Marmon, Vanda Marosi, Klaus F. X. Mayer, Steve Michel, Raja Sekhar Nandety, Kirby T. Nilsen, Edyta Paczos-Grzęda, Asher Pasha, Elena Prats, Nicholas J. Provart, Adriana Ravagnani, Robert W. Reid, Jessica A. Schlueter, Alan H. Schulman, Taner Z. Sen, Jaswinder Singh, Mehtab Singh, Nick Sirijovski, Nils Stein, Bruno Studer, Sirja Viitala, Shauna Vronces, Sean Walkowiak, Penghao Wang, Amanda J. Waters, Charlene P. Wight, Weikai Yan, Eric Yao, Xiao-Qi Zhang, Gaofeng Zhou, Zhou Zhou, Nicholas A. Tinker, Jason D. Fiedler, Chengdao Li, Peter J. Maughan, Manuel Spannagl, Martin Mascher

**Affiliations:** 1 Leibniz Institute of Plant Genetics and Crop Plant Research (IPK) Gatersleben, Seeland, Germany; 2 School of Life Sciences, Technical University of Munich, Freising, Germany; 3 Plant Genome and Systems Biology (PGSB), Helmholtz Center Munich–German Research Center for Environmental Health, Neuherberg, Germany; 4 Natural Resources Institute Finland (LUKE), Helsinki, Finland; 5 Plant and Wildlife Sciences, Brigham Young University, Provo, UT USA; 6 Ottawa Research and Development Centre, Agriculture and Agri-Food Canada, Ottawa, Ontario Canada; 7 Western Crop Genetics Alliance, Food Futures Institute and School of Agriculture, Murdoch University, Perth, Western Australia Australia; 8 Institute of Biotechnology, University of Helsinki, Helsinki, Finland; 9 Institut de Biologie Intégrative et des Systèmes, Université Laval, Québec City, Quebec Canada; 10 Institute for Sustainable Agriculture, CSIC, Córdoba, Spain; 11 Cereal Crops Improvement Research Unit, USDA-ARS, Edward T. Schafer Agricultural Research Center, Fargo, ND USA; 12 Department of Plant Science, University of Manitoba, Winnipeg, Manitoba Canada; 13 Molecular Plant Breeding, Institute of Agricultural Sciences, ETH Zurich, Zurich, Switzerland; 14 Arizona Genomics Institute, University of Arizona, Tucson, AZ USA; 15 Agriculture and Food Solutions, General Mills, Minneapolis, MN USA; 16 Small Grains and Potato Germplasm Research Unit, USDA-ARS, Aberdeen, ID USA; 17 Plant Gene Resources of Canada, Saskatoon Research and Development Centre, Agriculture and Agri-Food Canada, Saskatoon, Saskatchewan Canada; 18 Julius Kuehn Institute (JKI), Federal Research Centre for Cultivated Plants, Institute for Breeding Research on Agricultural Crops, Sanitz, Germany; 19 IBERS Gogerddan, Aberystwyth University, Aberystwyth, UK; 20 Centro de Biotecnología y Genómica de Plantas (CBGP), Universidad Politécnica de Madrid (UPM) & Instituto Nacional de Investigación y Tecnología Agraria y Alimentaria (INIA), Madrid, Spain; 21 R. W. Holley Center for Agriculture and Health, USDA-ARS, Ithaca, NY USA; 22 School of Integrative Plant Science, Cornell University, Ithaca, NY USA; 23 Department of Plant Science, McGill University, Sainte-Anne-de-Bellevue, Quebec Canada; 24 ScanOats Industrial Research Center, Department of Chemistry, Division of Pure and Applied Biochemistry, Lund University, Lund, Sweden; 25 Crop Improvement and Genetics Research Unit, USDA-ARS, Albany, CA USA; 26 Agriculture and Agri-Food Canada, Brandon, Manitoba Canada; 27 Institute of Plant Genetics, Breeding and Biotechnology, University of Life Sciences in Lublin, Lublin, Poland; 28 Department of Cell and Systems Biology and Centre for the Analysis of Genome Evolution and Function, University of Toronto, Toronto, Ontario Canada; 29 Department of Bioinformatics and Genomics, University of North Carolina at Charlotte, Charlotte, NC USA; 30 Viikki Plant Science Centre, Helsinki, Finland; 31 Department of Bioengineering, University of California, Berkeley, Berkeley, CA USA; 32 Institute of Agricultural and Nutritional Sciences, Martin Luther University Halle-Wittenberg, Halle, Germany; 33 Canadian Grain Commission, Winnipeg, Manitoba Canada; 34 School of Medical, Molecular and Forensic Sciences, Murdoch University, Perth, Western Australia Australia; 35 Research and Development Division, PepsiCo, Valhalla, NY USA; 36 Centre for Crop and Food Innovation, Food Futures Institute, Murdoch University, Perth, Western Australia Australia; 37 Department of Primary Industry and Regional Development, Government of Western Australia, Perth, Western Australia Australia; 38 College of Agronomy, Shandong Agricultural University, Tai’An, China; 39 German Centre for Integrative Biodiversity Research (iDiv) Halle-Jena-Leipzig, Leipzig, Germany; 40 Present Address: Global Oatly Science and Innovation Centre, Lund, Sweden

**Keywords:** Structural variation

Correction to: *Nature* 10.1038/s41586-025-09676-7 Published online 29 October 2025

In the version of the article initially published, the wrong caption was included for Extended Data Fig. 10. The correct caption is now available in the HTML and PDF versions of the article, and the original, incorrect caption is available for comparison in the Supplementary information accompanying this notice.

**Supplementary information** is available in the online version of this amendment.

## Supplementary information

Original, uncorrected Extended Data Fig. 10 caption

