# Supplementary material for: A pangenome and pantranscriptome of hexaploid oat
Source: Nature. 2025 Oct 29;649(8095):131–9. doi: 10.1038/s41586-025-09676-7 (PMC12727504; doi:10.1038/s41586-025-09676-7)
Supplement: Supplementary file 1 — Supplementary Figures 1–8 and Supplementary References. [file 41586_2025_9676_MOESM1_ESM.docx]

**Analysis of Gene Expression Changes at Translocation Breakpoints**

We assessed gene expression changes at translocation breakpoints by first identifying breakpoints in the GS7 genome through alignment of each oat line’s chromosomes to GS7 using Minimap2[^1^](https://paperpile.com/c/eXfrh9/et8v) and extracting 100 syntelogs flanking each breakpoint with GENESPACE[^2^](https://paperpile.com/c/eXfrh9/mglz). RNA-seq data from all lines were mapped to the GS7 transcriptome using Kallisto[^3^](https://paperpile.com/c/eXfrh9/N1Md), and differential gene expression analysis was performed with DESeq2[^4^](https://paperpile.com/c/eXfrh9/5iid), comparing expression across multiple tissues between lines carrying translocations and those without. To test whether regions surrounding breakpoints were enriched for differentially expressed genes relative to the rest of the chromosome, we applied a hypergeometric test with FDR correction (Benjamini-Hochberg, α = 0.05).

This revealed significant enrichment of DEGs near breakpoints on chromosomes 1A, 3C, and 7D (**Supplementary Fig. 1**), while the reciprocal translocation between 2A and 2C showed markedly fewer DEGs. Functional analyses highlighted enrichment for carbohydrate metabolism, particularly sucrose metabolism, along with key enzymatic activities (oxidoreductases and hydrolases) and regulatory components such as WAK/WAKL kinases, MADS/AGL transcription factors, and cytochrome P450s near breakpoints on chromosomes 1A, 1C, 2C, and 7D **(Supplementary Table 12)**. These results suggest that translocations can induce localized gene expression changes at their breakpoints, affecting metabolic and regulatory pathways while leaving genome-wide expression largely stable.


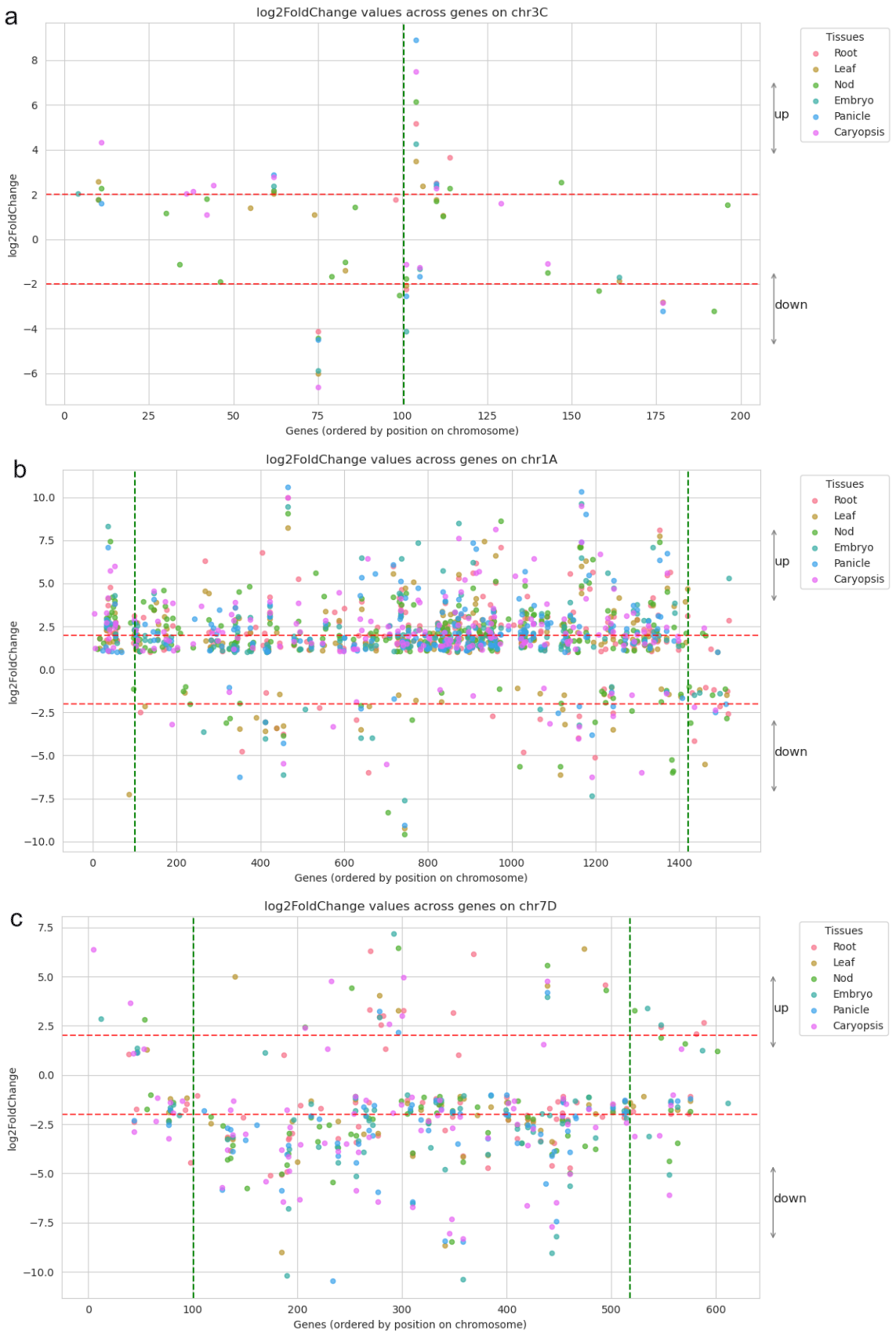


**Supplementary Fig. 1:** Differential gene expression (log2FoldChange) across genes ordered by chromosomal position for three translocated regions: **(a)** chr3C, **(b)** chr1A, and **(c)** chr7D. Each point represents the log2FoldChange of a gene in one of six tissues (Root, Leaf, Nodules, Embryo, Panicle, Caryopsis), colored as indicated. Horizontal red dashed lines denote log2FoldChange thresholds of ±2, marking genes with substantial up- or downregulation in translocated lines relative to non-translocated lines. Vertical green dashed lines indicate translocation breakpoints with flanking regions (100 genes upstream and downstream).

**Benchmarking of gene expression quantification methods in polyploid oat**

To evaluate the robustness of gene expression quantification in polyploid oat, we compared three widely used tools: Kallisto[^3^](https://paperpile.com/c/eXfrh9/N1Md), STAR[^5^](https://paperpile.com/c/eXfrh9/gnKs), and HISAT2[^6^](https://paperpile.com/c/eXfrh9/cZWV), —alongside the polyploid-specific classifier EAGLE-RC[^7^](https://paperpile.com/c/eXfrh9/gCH3). Kallisto was originally used in this study due to its computational efficiency and pseudoalignment approach, which probabilistically assigns multi-mapping reads and avoids alignment artefacts common in highly similar homeologous regions. However, given the complexity of the oat genome, we further assessed the consistency of subgenome-level expression patterns across methods.

EAGLE-RC, a specialized tool for polyploid expression analysis, was adapted for this analysis by modifying its source code, as its original implementation was hard-coded for wheat genomes and assumes no structural rearrangements or gene content exchange between subgenomes—assumptions that do not hold for oat’s mosaic genome structure[^8^](https://paperpile.com/c/eXfrh9/UjVS)

All four tools produced broadly consistent subgenome expression profiles. Boxplots of log-transformed transcripts per million (TPM) values for the A, C, and D subgenomes (**Supplementary Fig. 2**) showed similar distributions across methods, with Kallisto capturing subgenome-specific expression trends comparable to those observed with STAR, HISAT2, and EAGLE-RC.

To quantify concordance, Spearman correlations of gene-level TPM values were calculated between the four tools (**Supplementary Fig. 3**). Correlations exceeded 92% in all pairwise comparisons, indicating high agreement across methods. Notably, STAR, HISAT2, and EAGLE-RC—each relying on full alignment and downstream quantification—exhibited the strongest correlations among each other, while Kallisto showed slightly lower correlations relative to these alignment-based approaches. These differences likely reflect methodological contrasts: Kallisto’s pseudoalignment strategy avoids potential biases introduced during alignment, but inherently differs in read assignment compared to full aligners coupled with FeatureCounts[^9^](https://paperpile.com/c/eXfrh9/r6Md).

These results confirm that the subgenome expression patterns reported in the main study are robust across multiple quantification strategies, supporting the reliability of Kallisto in analyzing gene expression in complex polyploid genomes such as oat.


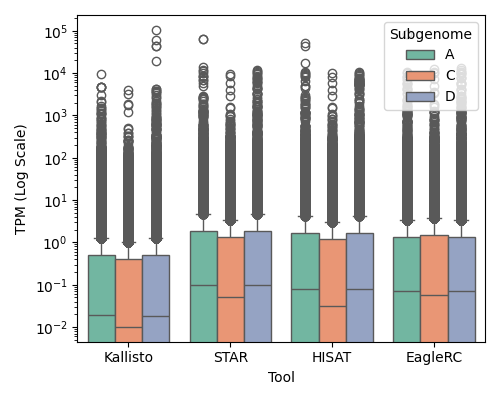


**Supplementary Fig. 2** Boxplot of log-transformed TPM values for the A, C, and D subgenomes, estimated using Kallisto, STAR, HISAT2, and EAGLE-RC.


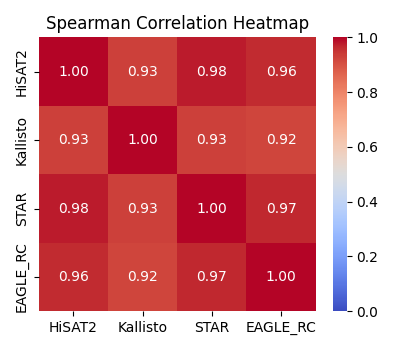


**Supplementary Fig. 3** Spearman correlation heatmap of gene expression levels (TPM) between Kallisto, STAR, HISAT2, and EAGLE-RC.


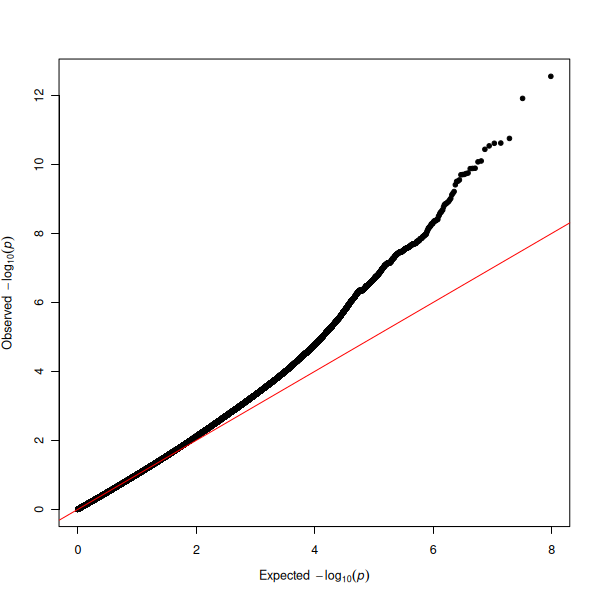


**Supplementary Fig. 4** QQ plot for kmerGWAS analysis based on the Ithaca 2010 heading date phenotype, demonstrating appropriate control of type I error and no inflation of test statistics.


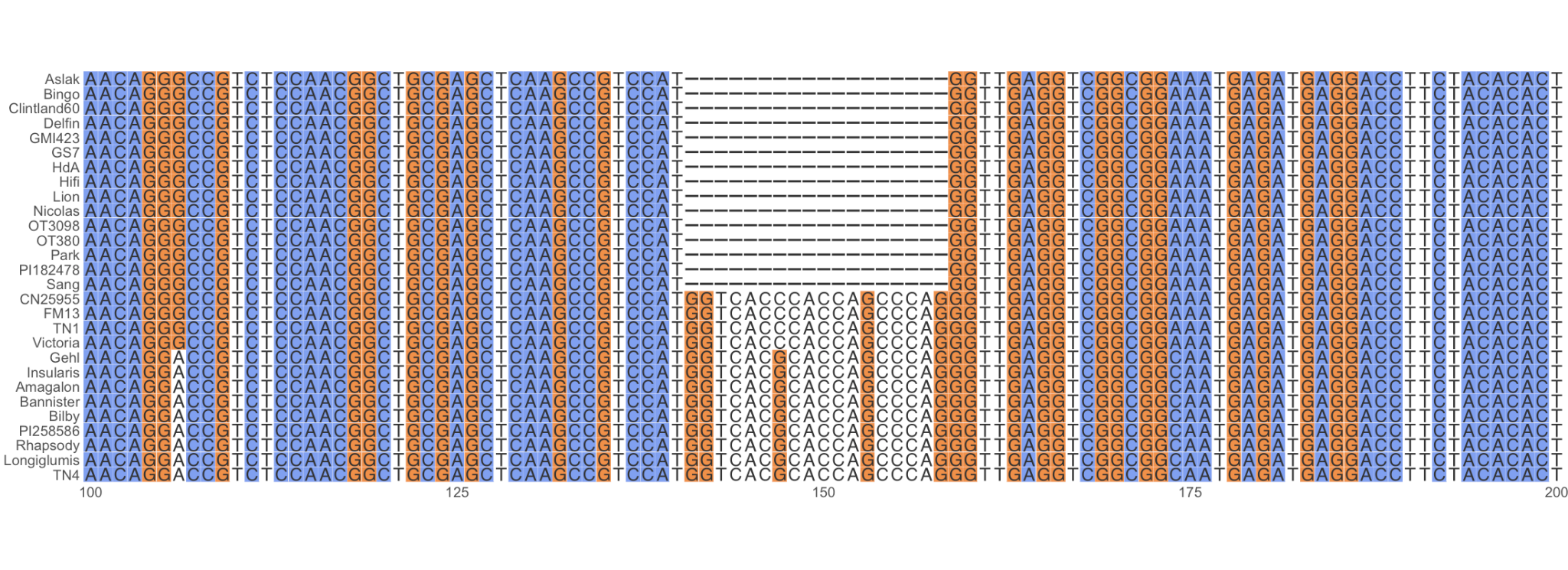


**Supplementary Fig. 5** Multiple sequence alignment of FT1-7D CDS from the pangenome assemblies showing a 18bp deletion.


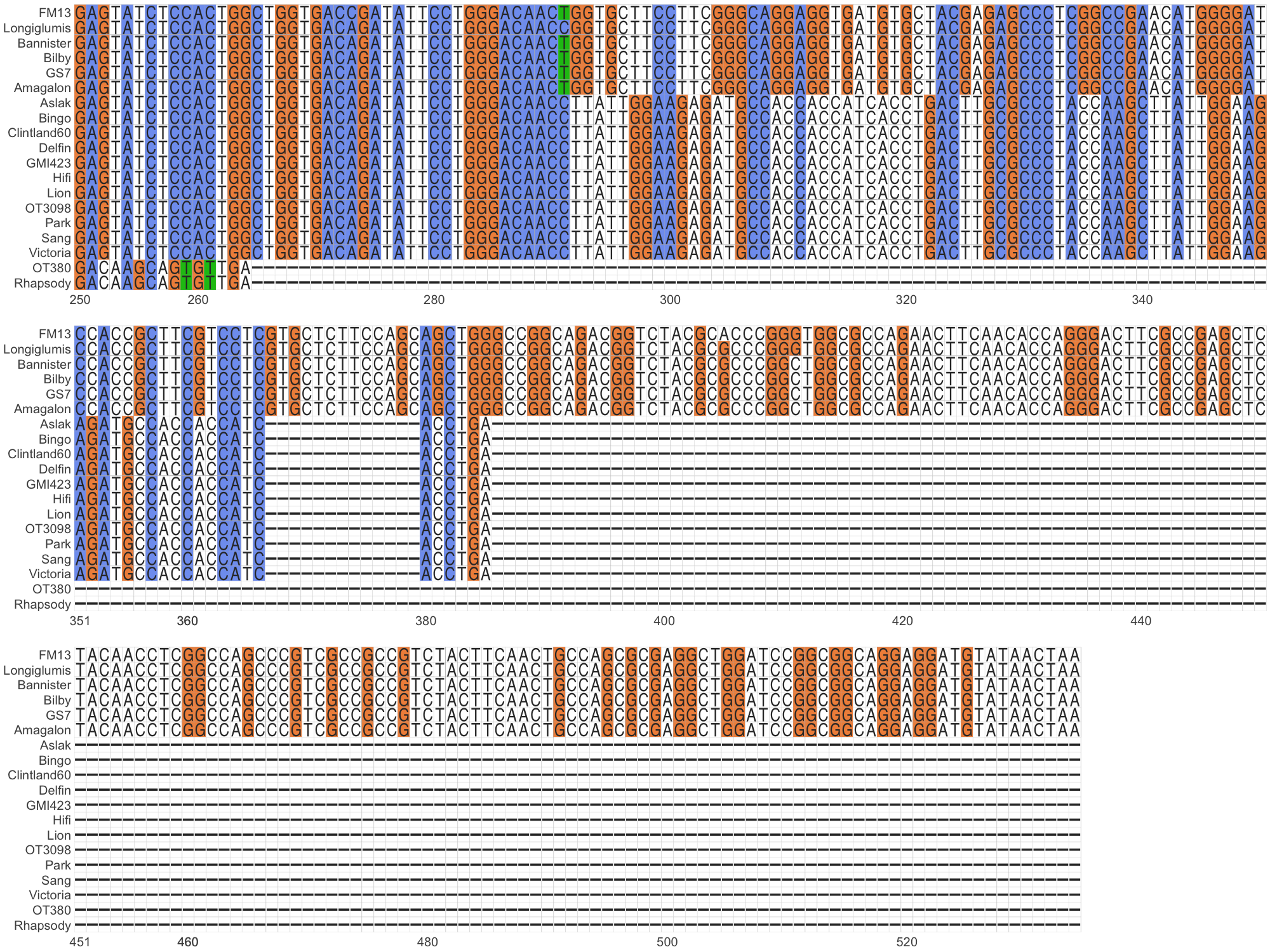


**Supplementary Fig. 6**. Multiple sequence alignment of FT1-7A CDS from the pangenome assemblies showing deletions at the end of the genes that cause a premature stop codon.


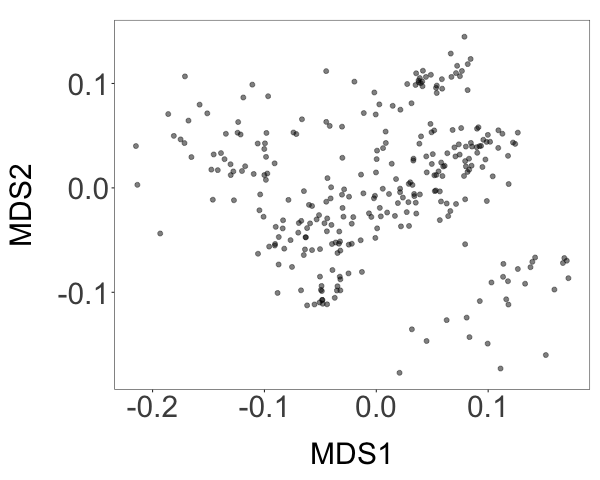


**Supplementary Fig. 7** MDS plot of 295 CORE lines, the analysis includes 67,368,800

SNPs from all chromosomes.

**Supplementary Fig. 8** PCA analysis of 142 Australian lines with and without the 2A/2C translocation event. The plot shows that the translocation does not affect the population structure.

**References**

1. [Li, H. (2018). Minimap2: pairwise alignment for nucleotide sequences. Bioinformatics *34*, 3094–3100.](http://paperpile.com/b/eXfrh9/et8v)

2. [Lovell, J.T., Sreedasyam, A., Schranz, M.E., Wilson, M., Carlson, J.W., Harkess, A., Emms, D., Goodstein, D.M., and Schmutz, J. (2022). GENESPACE tracks regions of interest and gene copy number variation across multiple genomes. Elife *11*. https://doi.org/](http://paperpile.com/b/eXfrh9/mglz)[10.7554/eLife.78526](http://dx.doi.org/10.7554/eLife.78526)[.](http://paperpile.com/b/eXfrh9/mglz)

3. [Bray, N.L., Pimentel, H., Melsted, P., and Pachter, L. (2016). Near-optimal probabilistic RNA-seq quantification. Nat. Biotechnol. *34*, 525–527.](http://paperpile.com/b/eXfrh9/N1Md)

4. [Love, M.I., Huber, W., and Anders, S. (2014). Moderated estimation of fold change and dispersion for RNA-seq data with DESeq2. Genome Biol. *15*, 550.](http://paperpile.com/b/eXfrh9/5iid)

5. [Dobin, A., Davis, C.A., Schlesinger, F., Drenkow, J., Zaleski, C., Jha, S., Batut, P., Chaisson, M., and Gingeras, T.R. (2013). STAR: ultrafast universal RNA-seq aligner. Bioinformatics *29*, 15–21.](http://paperpile.com/b/eXfrh9/gnKs)

6. [Kim, D., Paggi, J.M., Park, C., Bennett, C., and Salzberg, S.L. (2019). Graph-based genome alignment and genotyping with HISAT2 and HISAT-genotype. Nat. Biotechnol. *37*, 907–915.](http://paperpile.com/b/eXfrh9/cZWV)

7. [Kuo, T.C.Y., Hatakeyama, M., Tameshige, T., Shimizu, K.K., and Sese, J. (2020). Homeolog expression quantification methods for allopolyploids. Brief. Bioinform. *21*, 395–407.](http://paperpile.com/b/eXfrh9/gCH3)

8. [Kamal, N., Tsardakas Renhuldt, N., Bentzer, J., Gundlach, H., Haberer, G., Juhász, A., Lux, T., Bose, U., Tye-Din, J.A., Lang, D., et al. (2022). The mosaic oat genome gives insights into a uniquely healthy cereal crop. Nature *606*, 113–119.](http://paperpile.com/b/eXfrh9/UjVS)

9. [Liao, Y., Smyth, G.K., and Shi, W. (2014). featureCounts: an efficient general purpose program for assigning sequence reads to genomic features. Bioinformatics *30*, 923–930.](http://paperpile.com/b/eXfrh9/r6Md)
