# Supplementary material for: A pangenome and pantranscriptome of hexaploid oat
Source: Nature. 2025 Oct 29;649(8095):131–9. doi: 10.1038/s41586-025-09676-7 (PMC12727504; doi:10.1038/s41586-025-09676-7)
Supplement: Supplementary file 2 — Reporting Summary [file 41586_2025_9676_MOESM2_ESM.pdf]

nature portfolio

Corresponding author(s): Nicholas A. Tinker
Jason D. Fiedler
Chengdao Li
Peter J. Maughan
Manuel Spannagl
Martin Mascher

Last updated by author(s): 09/16/2025

Reporting Summary

Nature Portfolio wishes to improve the reproducibility of the work that we publish. This form provides structure for consistency and transparency in reporting. For further information on Nature Portfolio policies, see our Editorial Policies and the Editorial Policy Checklist.

Statistics

For all statistical analyses, confirm that the following items are present in the figure legend, table legend, main text, or Methods section.

n/a	Confirmed	
☐	☒	The exact sample size (*n*) for each experimental group/condition, given as a discrete number and unit of measurement
☐	☒	A statement on whether measurements were taken from distinct samples or whether the same sample was measured repeatedly
☐	☒	The statistical test(s) used AND whether they are one- or two-sided<br>*Only common tests should be described solely by name; describe more complex techniques in the Methods section.*
☒	☐	A description of all covariates tested
☐	☒	A description of any assumptions or corrections, such as tests of normality and adjustment for multiple comparisons
☐	☒	A full description of the statistical parameters including central tendency (e.g. means) or other basic estimates (e.g. regression coefficient) AND variation (e.g. standard deviation) or associated estimates of uncertainty (e.g. confidence intervals)
☐	☒	For null hypothesis testing, the test statistic (e.g. *F*, *t*, *r*) with confidence intervals, effect sizes, degrees of freedom and *P* value noted<br>*Give P values as exact values whenever suitable.*
☒	☐	For Bayesian analysis, information on the choice of priors and Markov chain Monte Carlo settings
☒	☐	For hierarchical and complex designs, identification of the appropriate level for tests and full reporting of outcomes
☐	☒	Estimates of effect sizes (e.g. Cohen's *d*, Pearson's *r*), indicating how they were calculated

Our web collection on statistics for biologists contains articles on many of the points above.

Software and code

Policy information about availability of computer code

Data collection	No software used for data collection.
Data analysis	Multiple published software packages were used in the analysis including: BBMap v37.93, Bcftools v1.12, bedtools v2.31.0, BFC v1.0, blast v2.14.0, blast v2.12, clusterProfiler v4.6, CRAM v3.1, cutadapt v3.3, Deseq2 , diamond v2.1.8, EVidenceModeler v1.1.1, Fastp v.0.24.1, GENESPACE v1.2.2, GenomeThreader v1.7.3, genometools-genometools v1.6.5, gffread v0.12.7, gmap v2018.07.04, HiRise pipeline v2.0.5, Kallisto, kmerGWAS v2, LiftoffTools v0.4.4, Mercator4 v6.0, mikado v2.3.4, Minia3 v3.2.0, minimap2 v2.26, minimap2 v2.1, Minimap2 v2.24, miniprot v0.11, MMSeqs2 v13, MSTmap, NGenomeSyn v1.41, Novosort v4.03.01, Orthofinder v2.5.5, PASApipeline v2.5.3, pfam database release 34, PLINK v1.9, portcullis v1.2.4, prodigal v2.6.3, prot-scriber v0.1.5, R statistical environment v3.5.1, samtools v1.17, samtools v1.13, samtools v1.16.1, SOAPDenovo2 v2.04-r240, star v2.7.10b, stringtie 2.2.1, TransDecoder v5.2.0, vmatch v2.3.0

For manuscripts utilizing custom algorithms or software that are central to the research but not yet described in published literature, software must be made available to editors and reviewers. We strongly encourage code deposition in a community repository (e.g. GitHub). See the Nature Portfolio guidelines for submitting code & software for further information.

Data

Policy information about availability of data

All manuscripts must include a data availability statement. This statement should provide the following information, where applicable:

- Accession codes, unique identifiers, or web links for publicly available datasets
- A description of any restrictions on data availability
- For clinical datasets or third party data, please ensure that the statement adheres to our policy

The data generated by the PanOat Consortium are made freely available and publicly accessible through deposition in public databases. Sequence data were deposited in the European Nucleotide Archive under project IDs PRJEB56828 (genome assembly raw data), PRJEB57570 (transcriptome sequencing) and PRJEB62778 (WGS resequencing data). Project IDs for individual assemblies and BioSample IDs for individual CORE genotypes are listed in Supplementary Tables 17 and 13, respectively. The annotation datasets are available for download from the USDA-ARS GrainGenes database17 at https://graingenes.org//GG3/content/panoat-data-download-page. This page also serves as a landing page for access not only to data but also to genome browser tools and BLAST services.

Research involving human participants, their data, or biological material

Policy information about studies with human participants or human data. See also policy information about sex, gender (identity/presentation), and sexual orientation and race, ethnicity and racism.

Reporting on sex and gender	Not applicable.
Reporting on race, ethnicity, or other socially relevant groupings	Not applicable.
Population characteristics	Not applicable.
Recruitment	Not applicable.
Ethics oversight	Not applicable.

Note that full information on the approval of the study protocol must also be provided in the manuscript.

Field-specific reporting

Please select the one below that is the best fit for your research. If you are not sure, read the appropriate sections before making your selection.

☒ Life sciences ☐ Behavioural & social sciences ☐ Ecological, evolutionary & environmental sciences

For a reference copy of the document with all sections, see nature.com/documents/nr-reporting-summary-flat.pdf

Life sciences study design

All studies must disclose on these points even when the disclosure is negative.

Sample size	Population structure of a diversity panel (CORE) was analyzed. Representative accessions were chosen.
Data exclusions	No data were excluded.
Replication	DNA/RNA was extracted from verified clones of the same genotypes.
Randomization	Genome assembly and analysis were conducted on a individual Avena ssp. plant, thus randomization is not necessary.
Blinding	Genome assembly and analysis were conducted on a individual Avena ssp. plant, thus randomization and/or blinding is not necessary.

Reporting for specific materials, systems and methods

We require information from authors about some types of materials, experimental systems and methods used in many studies. Here, indicate whether each material, system or method listed is relevant to your study. If you are not sure if a list item applies to your research, read the appropriate section before selecting a response.

Materials & experimental systems

n/a	Involved in the study	
☒	☐	Antibodies
☒	☐	Eukaryotic cell lines
☒	☐	Palaeontology and archaeology
☒	☐	Animals and other organisms
☒	☐	Clinical data
☒	☐	Dual use research of concern
☐	☒	Plants

Methods

n/a	Involved in the study	
☒	☐	ChIP-seq
☒	☐	Flow cytometry
☒	☐	MRI-based neuroimaging

Dual use research of concern

Policy information about dual use research of concern

Hazards

Could the accidental, deliberate or reckless misuse of agents or technologies generated in the work, or the application of information presented in the manuscript, pose a threat to:

No	Yes	
☒	☐	Public health
☒	☐	National security
☒	☐	Crops and/or livestock
☒	☐	Ecosystems
☒	☐	Any other significant area

Experiments of concern

Does the work involve any of these experiments of concern:

No	Yes	
☒	☐	Demonstrate how to render a vaccine ineffective
☒	☐	Confer resistance to therapeutically useful antibiotics or antiviral agents
☒	☐	Enhance the virulence of a pathogen or render a nonpathogen virulent
☒	☐	Increase transmissibility of a pathogen
☒	☐	Alter the host range of a pathogen
☒	☐	Enable evasion of diagnostic/detection modalities
☒	☐	Enable the weaponization of a biological agent or toxin
☒	☐	Any other potentially harmful combination of experiments and agents

Plants

Seed stocks	Seeds of the of pangenome panel and CORE population are available from German federal ex situ genebank at IPK Gatersleben.de OR USDA-ARS genebank https://www.ars-grin.gov/.
Novel plant genotypes	*Describe the methods by which all novel plant genotypes were produced. This includes those generated by transgenic approaches, gene editing, chemical/radiation-based mutagenesis and hybridization. For transgenic lines, describe the transformation method, the number of independent lines analyzed and the generation upon which experiments were performed. For gene-edited lines, describe the editor used, the endogenous sequence targeted for editing, the targeting guide RNA sequence (if applicable) and how the editor was applied.*
Authentication	*Describe any authentication procedures for each seed stock used or novel genotype generated. Describe any experiments used to assess the effect of a mutation and, where applicable, how potential secondary effects (e.g. second site T-DNA insertions, mosiacism, off-target gene editing) were examined.*
