# Supplementary material for: Author Correction: A pangenome and pantranscriptome of hexaploid oat
Source: Nature. 2025 Nov 20;648(8093):E5. doi: 10.1038/s41586-025-09919-7 (PMC12695643; doi:10.1038/s41586-025-09919-7)
Supplement: Supplementary file 1 — Original, uncorrected Extended Data Fig. 10 caption [file 41586_2025_9919_MOESM1_ESM.pdf]

nature portfolio

https://doi.org/10.1038/s41586-025-09919-7

Supplementary information

Author Correction: A pangenome and pantranscriptome of hexaploid oat

In the format provided by the authors and unedited

Original, uncorrected Extended Data Fig. 10 caption

a

Cross	1A	1C	1D	2A	2C	2D	3A	3C	3D	4A	4C	4D	5A	5C	5D	6A	6C	6D	7A	7C	7D
19S32	52:2:46<br>0.527	54:2:44<br>0.499	49:1:50<br>0.502	40:1:59<br>0.078	44:1:55<br>0.229	48:2:50<br>0.664	54:1:46<br>0.338	47:1:52<br>0.300	41:2:58<br>0.164	50:1:49<br>0.441	55:1:44<br>0.327	40:2:59<br>0.100	57:1:42<br>0.132	43:1:56<br>0.182	49:1:50<br>0.465	39:1:60<br>0.061	46:1:53<br>0.383	50:1:49<br>0.436	61:1:38<br>0.025	42:1:57<br>0.120	51:2:47<br>0.644
19S43	41:1:58<br>0.107	48:1:50<br>0.529	34:1:65<br>0.005	24:1:75<br>0.000	31:1:68<br>0.001	24:1:75<br>0.000	42:1:57<br>0.198	41:1:58<br>0.091	51:2:47<br>0.689	34:2:64<br>0.009	54:1:46<br>0.289	49:1:49<br>0.577	55:1:44<br>0.297	43:2:55<br>0.308	43:1:56<br>0.270	43:1:56<br>0.270	43:1:56<br>0.270	43:1:56<br>0.270	43:1:56<br>0.270	43:1:56<br>0.270	43:1:56<br>0.270
19S10	49:1:50<br>0.465	38:1:61<br>0.031	56:1:43<br>0.244	42:2:56<br>0.296	40:2:58<br>0.115	59:1:40<br>0.058	47:1:52<br>0.394	44:1:55<br>0.240	63:1:36<br>0.011	54:2:44<br>0.494	64:1:35<br>0.006	36:2:62<br>0.032	51:1:48<br>0.439	51:1:48<br>0.559	42:1:57<br>0.136	57:1:42<br>0.180	43:1:56<br>0.160	31:1:68<br>0.001	44:1:55<br>0.320	24:1:75<br>0.000	64:1:35<br>0.004
19S16	48:1:52<br>0.316	46:1:53<br>0.451	55:1:43<br>0.298	53:1:46<br>0.475	48:2:50<br>0.747	57:1:42<br>0.145	44:1:55<br>0.209	38:1:61<br>0.031	40:1:59<br>0.076	56:1:43<br>0.165	50:1:49<br>0.504	63:1:36<br>0.011	41:2:58<br>0.164	48:1:51<br>0.556	49:1:50<br>0.465	43:1:56<br>0.264	43:1:56<br>0.212	43:1:56<br>0.196	57:1:42<br>0.192	41:1:57<br>0.159	49:2:49<br>0.680
19S21	39:1:60<br>0.037	56:1:42<br>0.207	60:1:39<br>0.039	24:1:75<br>0.000	31:1:68<br>0.001	34:1:65<br>0.005	41:1:58<br>0.075	31:1:68<br>0.001	41:2:58<br>0.170	51:1:48<br>0.439	37:1:62<br>0.022	31:1:68<br>0.001	48:1:51<br>0.365	41:1:58<br>0.084	34:1:65<br>0.005	66:1:33<br>0.002	50:1:49<br>0.610	58:2:41<br>0.155	63:2:35<br>0.017	63:1:36<br>0.010	46:2:52<br>0.727
19S24	49:1:50<br>0.609	52:1:47<br>0.481	34:1:65<br>0.005	57:2:41<br>0.189	61:1:38<br>0.035	43:1:56<br>0.236	45:1:54<br>0.301	63:1:36<br>0.011	63:1:36<br>0.011	49:1:50<br>0.611	44:1:54<br>0.359	51:1:48<br>0.420	45:1:54<br>0.238	58:2:40<br>0.153	51:1:48<br>0.389	62:1:37<br>0.020	62:1:37<br>0.023	24:1:75<br>0.000	62:1:37<br>0.029	24:1:75<br>0.000	47:1:52<br>0.364
19S28	44:1:54<br>0.348	59:2:39<br>0.105	60:2:39<br>0.069	54:1:45<br>0.304	49:1:49<br>0.577	48:2:51<br>0.743	46:1:53<br>0.399	47:2:51<br>0.696	60:2:38<br>0.054	47:1:52<br>0.319	45:1:54<br>0.313	24:1:75<br>0.000	55:1:44<br>0.271	57:1:42<br>0.180	46:1:53<br>0.355	41:1:58<br>0.098	42:1:57<br>0.162	56:3:42<br>0.363	41:1:58<br>0.091	41:2:58<br>0.170	58:1:41<br>0.123
19S29	24:1:75<br>0.000	66:1:33<br>0.002	24:1:75<br>0.000	24:1:75<br>0.000	24:1:75<br>0.000	58:0:41<br>0.066	24:1:75<br>0.000	24:1:75<br>0.000	24:1:75<br>0.000	42:1:57<br>0.113	24:1:75<br>0.000	24:1:75<br>0.000	24:1:75<br>0.000	51:1:48<br>0.483	24:1:75<br>0.000	63:1:36<br>0.011	24:1:75<br>0.000	24:1:75<br>0.000	58:0:41<br>0.066	38:1:61<br>0.027	24:1:75<br>0.000
19S36	45:2:54<br>0.448	44:1:55<br>0.294	62:2:37<br>0.026	54:1:46<br>0.338	43:1:56<br>0.204	56:1:43<br>0.252	54:2:44<br>0.398	45:1:54<br>0.372	50:2:48<br>0.629	64:1:35<br>0.007	59:1:40<br>0.073	62:1:37<br>0.023	31:1:68<br>0.001	60:1:39<br>0.044	45:2:53<br>0.464	36:1:63<br>0.013	45:1:54<br>0.313	42:1:56<br>0.216	49:1:50<br>0.406	50:2:48<br>0.732	24:1:75<br>0.000
19S4	56:2:42<br>0.232	42:2:56<br>0.279	62:2:37<br>0.026	38:2:61<br>0.041	24:1:75<br>0.000	24:1:75<br>0.000	39:1:60<br>0.049	24:1:75<br>0.000	46:2:51<br>0.824	48:2:50<br>0.730	52:1:47<br>0.481	62:1:37<br>0.029	54:1:45<br>0.370	50:1:48<br>0.561	60:2:38<br>0.060	38:1:61<br>0.036	60:1:39<br>0.039	42:1:57<br>0.198	54:2:45<br>0.466	24:1:75<br>0.000	49:2:49<br>0.647
19S5	39:1:60<br>0.063	65:1:34<br>0.003	55:1:44<br>0.305	39:2:59<br>0.094	54:1:45<br>0.272	24:1:75<br>0.000	62:1:37<br>0.029	58:1:41<br>0.131	45:1:54<br>0.369	50:1:49<br>0.507	31:1:68<br>0.001	64:1:35<br>0.004	24:1:75<br>0.000	57:1:42<br>0.192	47:1:52<br>0.462	38:1:61<br>0.027	46:1:52<br>0.445	56:1:43<br>0.267	45:2:53<br>0.468	48:2:50<br>0.672	60:1:40<br>0.051
19S7	58:1:41<br>0.123	45:1:54<br>0.377	55:2:43<br>0.326	24:1:75<br>0.000	49:2:50<br>0.709	44:1:55<br>0.320	42:1:57<br>0.154	48:2:50<br>0.756	48:1:51<br>0.576	55:1:44<br>0.275	38:1:61<br>0.043	56:1:43<br>0.267	49:1:50<br>0.502	52:1:46<br>0.456	43:1:56<br>0.270	48:1:51<br>0.540	46:1:52<br>0.511	60:1:39<br>0.039	53:2:45<br>0.535	40:1:58<br>0.102	63:2:35<br>0.012
19S8	42:1:57<br>0.147	31:1:68<br>0.001	41:1:58<br>0.084	63:2:35<br>0.017	49:2:50<br>0.777	66:1:33<br>0.002	60:1:39<br>0.044	38:1:61<br>0.036	24:1:75<br>0.000	39:1:60<br>0.049	39:2:59<br>0.087	24:1:75<br>0.000	59:1:40<br>0.058	24:1:75<br>0.000	47:1:52<br>0.462	44:1:55<br>0.209	48:1:51<br>0.540	52:2:47<br>0.658	52:1:47<br>0.497	50:1:49<br>0.509	54:1:45<br>0.404

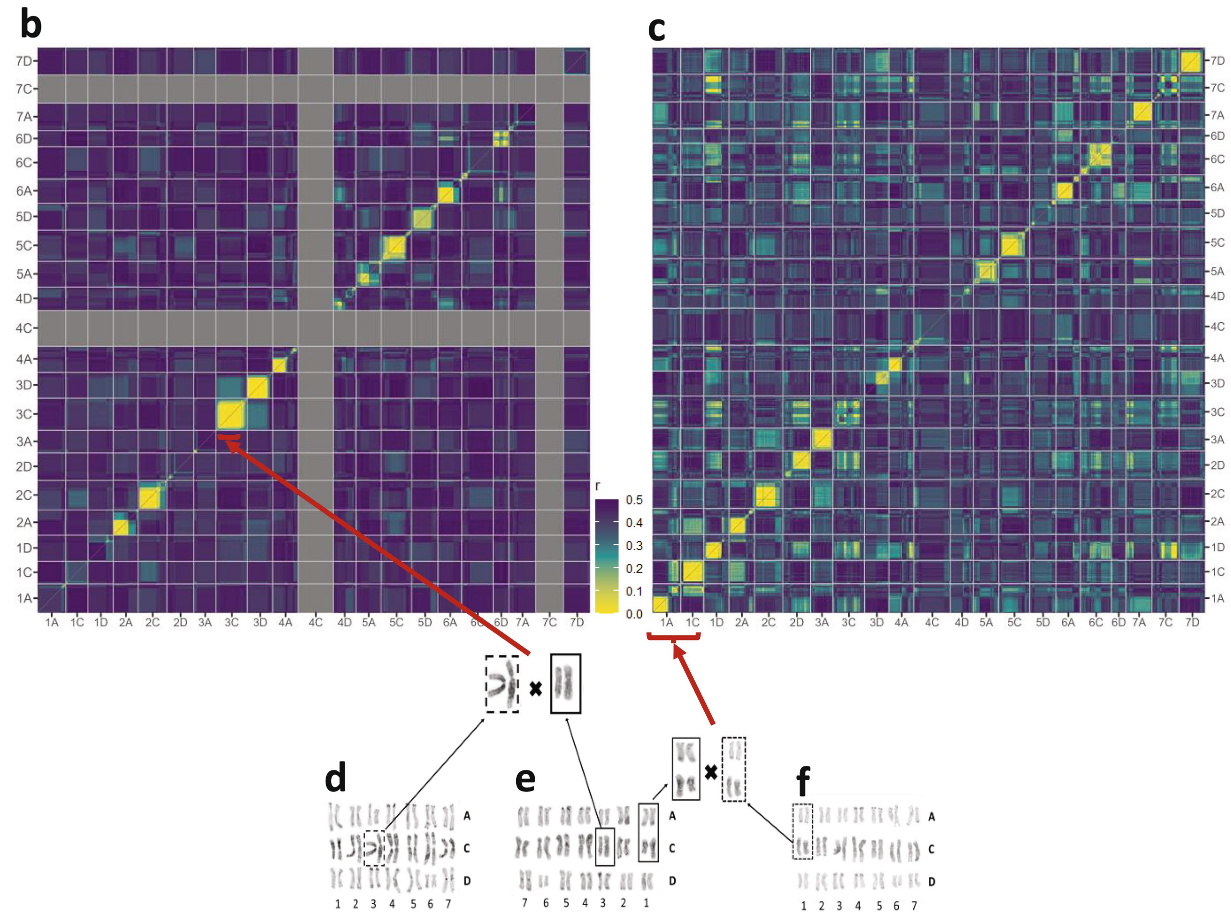

Extended Data Fig. 10 | See next page for caption.

Extended Data Fig. 10 | Large-scale chromosomal rearrangements shaped segregation and recombination patterns in the progenies of 13 crosses made at the Ottawa Research and Development Centre, AAFC, in 2019. The parental lines were selected for their excellent trait profiles and adaptation to different Canadian environments. Progenies from these crosses were genotyped for genomic selection at the F6 generation. Two populations (19S29 and 19S43) out of 13 exhibited unusual segregation ratios and recombination patterns. The progenies of the 19S43 population and those from the half-sib cross 19S32, which share the parent OA1613-5, displayed drastically different segregation ratios and patterns of recombination shown as recombination fractions heat maps between all pairs of markers ranging from purple (r = 0.5) to yellow (r = 0). **a**, The 19S32 progenies (n = 192) showed the expected segregation for biparental RILs at the F6 generation (AA:44.9, AB:0.9, BB:54.2). **b**, In contrast, the 19S43 progenies (n = 280) showed distorted segregation (AA:72.2, AB:1.2, BB:26.6). Chromosomes 1 A and 1 C of the 19S43 progenies exhibited suppressed recombination and a pattern typical of pseudo-linkage between 1A/1C, along with suppressed recombination on chromosome 7D, similar to the patterns reported by Tinker et al.[31]. Additional suppressed chromosomes include 2C, 3A, 3C, and 4D. The 19S32 population also exhibited some suppressed recombination, but only on chromosomes 2C and 3C. Karyotypes of the three parental lines confirmed they have a complete set of 21 chromosome pairs. **c**, The karyotype of OA1623-2, the female parent of 19S32, confirmed the presence of a heterozygous inversion on 3C and the common translocation on 1A. **d**, The karyotype of OA1613-5, the pollen donor for the two crosses, shows a homozygous 3C inversion (non-ancestral) and the common 1A/1C translocation, confirming the suppressed recombination in 3C and the expected patterns recombination in 1A and 1C of 19S32. **e**, The karyotype of OA1568-6, the female parent of 19S43, shows a pair of ancestral - non-translocated 1A chromosomes, confirming the pseudo-linkage patterns of recombination in crosses involving parents with and without the 1A/1C translocation, and suppressed recombination on 7D. The co-occurrence of chromosomal aberrations potentially explains the distorted segregation observed in this cross. All 13 crosses exhibited suppressed recombination in at least one of their chromosomes, similar to that seen in 19S32 (3C) or 19S43 (7D). The following chromosomes showed recombination suppression in three or more of the 13 populations: 1C, 2C, 3C, 4C, 5A, 7C and 7D.
